# Supplementary material for: Assessment of different factors on the influence of glass wool concentration for detection of main swine viruses in water samples
Source: PeerJ. 2023 Oct 4;11:e16171. doi: 10.7717/peerj.16171 (PMC10559894; doi:10.7717/peerj.16171)
Supplement: Supplemental Information 1 [file peerj-11-16171-s001.docx]

In the revised manuscript, the name of Huanchun Chen has been removed from the author list as he could not verify his email before the deadline when the submission of the revised manuscript was required. However, his contribution to the revision of the manuscript has been acknowledged in the “Acknowledgement” section.
