# Supplementary material for: Assessment of different factors on the influence of glass wool concentration for detection of main swine viruses in water samples
Source: PeerJ. 2023 Oct 4;11:e16171. doi: 10.7717/peerj.16171 (PMC10559894; doi:10.7717/peerj.16171)
Supplement: Supplemental Information 2 — In order to facilitate synchronous detection, after debugging and optimization, the qPCR reaction procedures of the four viruses were adjusted to be consistent after optimization: started with a hot start polymerase activation step for 5 min at 95 °C, followed by 40 cycles of 15 s at 95 °C and 1 min at 60 °C. [file peerj-11-16171-s002.docx]

**Table S1 Primers and protocols for qPCR detection of different viruses**

| Virus type | Primer（5-3） | Probe（5-3） | Reaction system |
| --- | --- | --- | --- |
| PRV-gH | F: ACGCTCGGCTTCCTCTCC  R: GGTAGTCGTCGCTCTCGTG | FAM-TCGCGCATCGTCTGGTGCAT-BHQ1 | PF 0.6μmol/L, PR 0.6μmol/L  Probe 0.3μmol/L, Mix 10μL  Template 2μL, Water add to 20μL |
| PRV-gE | F: GCTGTACGTGCTCGTGAT  R: TCAGCTCCTTGATGACCGTGA | FAM-CACAACGGCCACGTCGCCACCTG-BHQ1 | PF 0.6μmol/L, PR 0.6μmol/L  Probe 0.3μmol/L, Mix 10μL  Template 2μL, Water add to 20μL |
| ASFV | F: AACGCGTTCGCTTTTCG  R: CATCGTGGTGGTTATTGTTGGT | FAM-ACGTGTCCATAAAACGCAGGTGACCC-BHQ1 | PF 0.4μmol/L, PR 0.4μmol/L  Probe 0.3μmol/L, Mix 10μL  Template 2μL,Water add to 20μL |
| PEDV | F：CGTACAGGTAAGTCAATTAC  R：GATGAAGCATTGACTGAA | FAM-TTCGTCACAGTCGCCAAGG-BHQ1 | PF 0.6μmol/L, PR 0.6μmol/L  Probe 0. 4μmol/L, Mix 10μL  Template 2μL, Water add to 20μL |

In order to facilitate synchronous detection, after debugging and optimization, the qPCR reaction procedures of the four viruses were adjusted to be consistent after optimization: started with a hot start polymerase activation step for 5 min at 95°C, followed by 40 cycles of 15s at 95°C and 1 min at 60°C.
